# Murine gammaherpesvirus-68 ORF38 encodes a tegument protein and is packaged into virions during secondary envelopment

**DOI:** 10.1007/s13238-013-0005-0

**Published:** 2014-01-29

**Authors:** Sheng Shen, Haitao Guo, Hongyu Deng

**Affiliations:** 1 Chinese Academy of Sciences Key Laboratory of Infection and Immunity, Institute of Biophysics, Chinese Academy of Sciences, Beijing, 100101 China; 2 University of Chinese Academy of Sciences, Beijing, 100049 China

**Keywords:** gammaherpesvirus, tegument protein, ORF38, ORF33, TGN

## Abstract

Tegument is the unique structure of a herpesvirion which occupies the space between nucleocapsid and envelope. Accumulating data have indicated that interactions among tegument proteins play a key role in virion morphogenesis. Morphogenesis of gammaherpesviruses including Kaposi’s sarcoma-associated herpesvirus (KSHV) and Epstein-Barr virus (EBV) is poorly understood due to the lack of efficient de novo lytic replication in cell culture. Murine gammaherpesvirus-68 (MHV-68) is genetically related to these two human herpesviruses and serves as an effective model to study the lytic replication of gammaherpesviruses. We previously showed that ORF33 of MHV-68 encodes a tegument protein and plays an essential role in virion maturation in the cytoplasm. However, the molecular mechanism of how ORF33 participates in virion morphogenesis has not been elucidated. In this study we demonstrated that ORF38 of MHV-68 is also a tegument protein and is localized to cytoplasmic compartments during both transient transfection and viral infection. Immuno-gold labeling assay showed that ORF38 is only present on virions that have entered the cytoplasmic vesicles, indicating that ORF38 is packaged into virions during secondary envelopment. We further showed that ORF38 co-localizes with ORF33 during viral infection; therefore, the interaction between ORF38 and ORF33 is conserved among herpesviruses. Notably, we found that although ORF33 by itself is distributed in both the nucleus and the cytoplasm, in the presence of ORF38, ORF33 is co-localized to trans-Golgi network (TGN), a site where secondary envelopment takes place.

## Introduction

The herpesviridae is a family of large DNA viruses. Based on genome structure and biological properties, herpesviruses are classified into three subfamilies, *Alphaherpesvirinae*, *Betaherpesvirinae* and *Gammaherpesvirinae*. Gammaherpesviruses are also called tumor-associated herpesviruses. Epstein-Barr virus (EBV) and Kaposi’s sarcoma-associated herpesvirus (KSHV), two human gammaherpesviruses, are causative agents of several malignancies, such as lymphomas, nasopharyngeal carcinoma and Kaposi’s sarcoma (Soulier et al., 1995; Ganem, 2006; Kutok and Wang, 2006). Like other herpesviruses, EBV and KSHV are characterized as having two distinct phases in their life cycle: lytic replication and latency (Sunil-Chandra et al., 1992; Decker et al., 1996a, 1996b). Studies have shown that both lytic replication and latency play important roles in tumorigenesis (Bais et al., 1998; Martin et al., 1999; Stine et al., 2000; Blackman and Flano, 2002; Grundhoff and Ganem, 2004; Stevenson and Efstathiou, 2005).

A significant event during lytic replication is the formation of mature virions which are released into extracellular space to initiate new infection cycles in neighboring uninfected cells. A mature herpesvirion consists of a DNA genome, an icosahedral capsid, a thick tegument and an envelope containing lipid bilayer spiked with viral glycoproteins (Dai et al., 2008). Morphogenesis of a herpesvirion is a complicated multiple-step process. Briefly, it begins with the auto-assembly of capsid proteins into a capsid in the nucleus. After encapsidation of virus genome, a nucleocapsid travels to the inner nuclear membrane where it acquires primary envelope and enters into the perinuclear space. Nuclear egress or “de-envelopment” is accomplished by fusion of the primary envelope with the outer nuclear membrane and release of the nucleocapsid into the cytoplasm. The nucleocapsid acquires most tegument proteins and envelope proteins in the cytoplasm and completes its maturation at trans-Golgi network (TGN)-derived vesicles, a process called “secondary envelopment”. A mature virion is finally released into the extracellular space through fusion of the vesicles with the cellular membrane (Mettenleiter et al., 2006; Guo et al., 2010).

Until now, studies on morphogenesis of herpesviruses have mostly focused on alphaherpesviruses such as herpes simplex virus-1 (HSV-1) and to a less extent on beta-herpesviruses such as human cytomegalovirus (HCMV) (Mettenleiter et al., 2009). Little is known about the morphogenesis of gammaherpesviruses due to the lack of efficient lytic replication of KSHV and EBV in cell culture. Murine gammaherpesvirus-68 (MHV-68) is genetically related to these two human herpesviruses and replicates robustly in cell culture, presenting an effective model to study the de novo infection process of gammaherpesviruses (Efstathiou et al., 1990; Simas and Efstathiou, 1998). Using multiple approaches, we previously showed that MHV-68 ORF33 is a tegument protein that is essential for virion morphogenesis (Guo et al., 2009). However, the molecular mechanism of ORF33 involved in virion morphogenesis remained unclear. ORF33 is a conserved tegument protein in herpesvirus family. Studies on its homologues in HSV-1 and HCMV (UL16 and UL94, respectively) showed that they both interact with another conserved tegument protein, designated as UL11 in HSV-1 and UL99 in HCMV, and that both interactions are involved in virion maturation in the cytoplasm (Yeh et al., 2008; Phillips et al., 2012). The homologue of UL11 in MHV-68 is ORF38. A previous study on genome-wide protein interaction network of MHV-68 has showed that ORF33 interacts with ORF38 (Lee et al., 2011); however, the role of ORF38 in MHV-68 morphogenesis has not been characterized.

In this study, we first demonstrated that MHV-68 ORF38 is a tegument protein and that it is localized to cytoplasmic compartments during both transient transfection and viral infection. We further showed that ORF38 is packaged into virions within cytoplasmic vesicles during secondary envelopment. In addition, we showed co-localization of ORF38 and ORF33 during viral infection. Notably, we found that although ORF33 by itself is distributed in both the nucleus and the cytoplasm, its interaction with ORF38 makes it localize to TGN, a site where secondary envelopment takes place.

## Results

### MHV-68 *ORF38* encodes a tegument protein

To facilitate the characterization of MHV-68 ORF38, we first expressed and purified His-tagged ORF38 in *E. coli* and used it to generate a rabbit polyclonal antibody. Lysate from BHK-21 cells infected with MHV-68 was examined by Western blotting analysis to test the specificity of the antibody. A 12-kDa protein was detected in virus infected BHK cells (Fig. 1A, lane 1) but not in mock infected cells (Fig. 1A, lane 2). In contrast, no band was detected from either MHV-68-infected or mock-infected cells by preimmune rabbit serum (data not shown).

**Figure 1:**
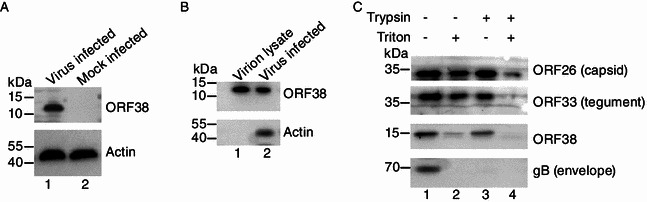
MHV-68 *ORF38* encodes a tegument protein. (A) *ORF38* is expressed during MHV-68 lytic infection. BHK cells were infected with WT MHV-68 at an MOI of 3. At 24 hpi, cells were lysed and subjected to Western blotting analysis with an anti-ORF38 polyclonal antibody or a monoclonal antibody specific for β-actin. Lysate from mock infected cells was used as a negative control. (B) ORF38 is a component of mature MHV-68 virions. Mature virions purified by gradient centrifugation were resolved via SDS-PAGE and analyzed using the anti-ORF38 polyclonal antibody. Lysate from virus infected BHK cells was used as a positive control. (C) Sensitivity of ORF38 to detergent and trypsin treatment. The purified virions were treated with trypsin or detergent or both, as described in MATERIALS AND METHODS. The resulting tegument-nucleocapsid complexes were subjected to Western blotting analysis individually using anti-ORF26, anti-ORF33, anti-ORF38 and anti-gB polyclonal antibodies

The MHV-68 *ORF38* encodes a protein of 75 aa and shares 27% amino acid sequence identity to its homologue in EBV (named BBLF1), which was identified as a component of the virion-associated proteins (Johannsen et al., 2004). The homologues of ORF38 in alpha- and beta-herpesvirus family were also reported to be virion-associated tegument proteins (Kattenhorn et al., 2004; Loret et al., 2008). Therefore, it is very likely that MHV-68 ORF38 is also a virion protein; however, a previous mass spectrometric analysis of purified MHV-68 virions did not report the identification of ORF38 (Bortz et al., 2003). We therefore decided to first determine whether ORF38 is associated with MHV-68 virions.

Mature MHV-68 virions were harvested from supernatant of MHV-68-infected cells and purified through gradient ultracentrifugation. Virions were lysed, resolved on SDS-PAGE, and analyzed by Western blotting, using the anti-ORF38 polyclonal antibody. A 12-kDa protein, consistent with the molecular weight of ORF38 found in MHV-68-infected cell lysate (Fig. 1B, lane 2), was detected in virion lysate (Fig. 1B, lane 1), demonstrating that ORF38 protein is indeed associated with viral particles. We next performed trypsin and detergent treatment experiment, a classical assay to determine the detailed localization of a virion protein within the viral particles (Bortz et al., 2003), and compared the sensitivity of ORF38 to trypsin and/or detergent treatment to that of known capsid protein ORF26, tegument protein ORF33, and envelope protein glycoprotein B (gB). As expected, gB was sensitive to trypsin in both the presence and the absence of detergent (Fig. 1C, bottom panel) whereas capsid protein ORF26 was resistant to trypsin and/or detergent treatment (Fig. 1C, top panel). Similar to ORF33, ORF38 was sensitive to trypsin treatment in the presence of detergent (Fig. 1C, lane 4). However, unlike ORF33, ORF38 was partially sensitive to detergent in the absence of trypsin (Fig. 1C, lane 2). As ORF33 has been identified as a tegument protein of MHV-68 that is associated tightly with the capsid (Guo et al., 2009), these data demonstrated that ORF38 is a tegument protein but binds less strongly to capsid than does ORF33.

### MHV-68 ORF38 localizes to trans-Golgi network (TGN)

The fact that ORF38 binds loosely to capsid suggests that ORF38 may be an outer tegument protein and participate in the secondary envelopment of viral particles. As the function of a protein is inevitably linked to its localization within the cell, we examined the subcellular localization of ORF38. Cells were infected with MHV-68 and indirect immunofluorescence assay was performed using the anti-ORF38 antibody. As shown in Fig. 2A, ORF38 mainly localized to the cytoplasm, with a predominant distribution in the juxtanuclear space. To further define the subcellular localization of ORF38, we investigated the co-localization of ORF38 with a panel of known protein markers for subcellular compartments. These markers, TGN38 for TGN, EEA1 for early endosome, LAMP1 for late endosome, and VPS37C for multivesicular bodies (MVB), respectively, are all fused to GFP. To facilitate the analysis, we first constructed a plasmid that expresses ORF38 fused to the N-terminus of RFP (named pDsRed-38). We then conducted co-transfection experiment with pDsRed-38 and a plasmid encoding one of the markers. As shown in Fig. 2B, ORF38 displayed partial co-localization with the TGN marker. Significant signal overlap was also observed between ORF38 and the late endosome marker. In contrast, minimal co-localization was observed between ORF38 and the early endosome marker or the MVB marker. As herpesvirus secondary envelopment is believed to occur at the TGN (Mettenleiter et al., 2009), the localization of ORF38 in TGN is consistent with its potential role in secondary envelopment during virion morphogenesis.

**Figure 2:**
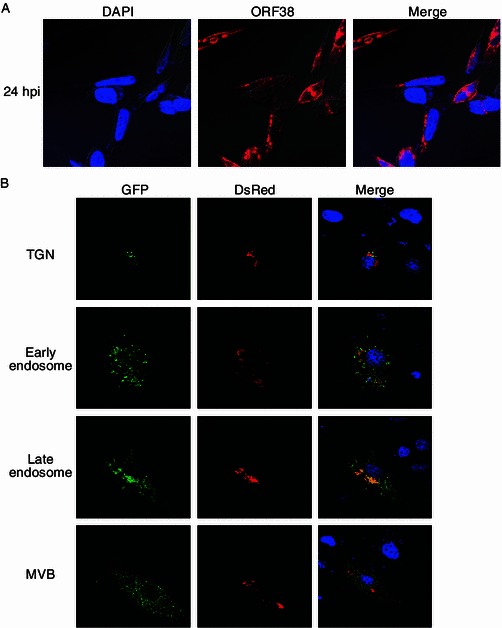
Subcellular localization of MHV-68 ORF38. (A) ORF38 localizes to the cytoplasm. Vero cells were infected with WT MHV-68 at an MOI of 3. At 24 hpi, cells were fixed for indirect immunofluorescence analysis using the anti-ORF38 polyclonal antibody. (B) Co-localization of ORF38 with different cytoplasmic organelles. Vero cells were cotransfected with pDsRed-38 plus pTGN-38 GFP or pEEA1-GFP or pLAMP1-GFP or pVPS37C-GFP. At 24 h posttransfection, cells were fixed, stained with DAPI, and examined with a confocal laser microscope

### ORF38 is packaged into virions during secondary envelopment

To determine the role of ORF38 in MHV-68 virion morphogenesis, we next performed an immuno-gold labeling assay to examine at which step of virion morphogenesis ORF38 is packaged into virions. Cells were infected with MHV-68, fixed and embedded. Samples were processed and ultrathin sections were obtained. The sections were immunolabeled with either the anti-ORF38 rabbit polyclonal antibody or the rabbit IgG as a negative control, followed by a secondary antibody conjugated with 5-nm gold particles. In the negative control sample treated with rabbit IgG, no gold particle was found on virions (Fig. 3A). In the sample treated with the anti-ORF38 antibody, viral particles were readily found in the nucleus, however, no gold particle was detected either on the viral particles or in other areas of the nucleus (Fig. 3B), consistent with the cytoplasmic localization of ORF38 as demonstrated by our immunofluorescence data (Fig. 2A). In contrast, abundant ORF38 was present around or within vesicles in the cytoplasm where virions were undergoing secondary envelopment (Fig. 3C and 3D). Notably, ORF38 was only present on virions that had entered vesicles but not on virions outside the vesicles (Fig. 3D). These data clearly indicated that the tegument protein ORF38 is packaged into virions during secondary envelopment.

**Figure 3:**
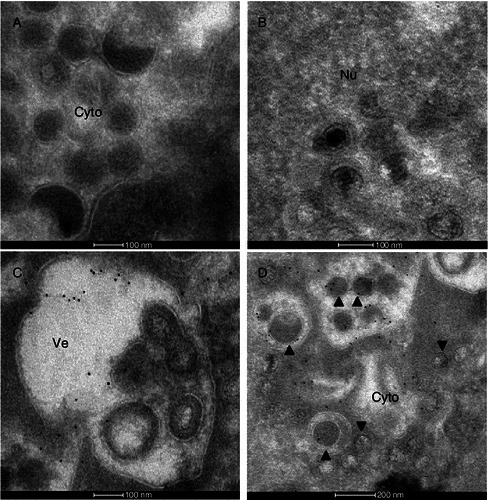
Immuno-gold labeling of ORF38 in MHV-68 infected cells. BHK cells were infected with WT MHV-68 at an MOI of 5. Ultrathin sections were immunolabeled with rabbit IgG as a negative control (A) or anti-ORF38 rabbit polyclonal antibody (B–D), followed by a secondary antibody conjugated with 5-nm gold particles. No gold particle was found in samples treated with rabbit IgG (A). ORF38 was not detected in the nucleus (Nu) of virus infected cells (B). In the cytoplasm (Cy), ORF38 was found around and within the vesicles (C and D) as well as on virions within the vesicles (▲) (D) but not found on virions outside the vesicles (▼)(D). Bars: A, B and C, 100 nm; D, 200 nm

### ORF38 interacts with ORF33

Tegumentation is an important stage of morphogenesis for a herpesvirus particle and involves a complex and dynamic process of protein-protein interactions. We therefore tested the interaction of ORF38 with other tegument proteins and found out that ORF38 interacts with ORF33, consistent with a previous report (Lee et al., 2011). Briefly, coimmunoprecipitation (co-IP) experiment was performed with cell lysates from 293T cells transiently transfected with either a plasmid expressing HA-tagged ORF33 (HA-33) or a plasmid expressing 3xFLAG-CBP-tagged ORF38 (TAG-38) or both. As shown in Fig. 4A, ORF38 interacted with ORF33 in the absence of other viral proteins. To confirm that the interaction between ORF33 and ORF38 exists in the context of viral infection, we next determined the relative localization of these two proteins during viral infection. Cells were infected with MHV-68 and indirect immunofluorescence assay was performed with the anti-ORF38 antibody and an anti-ORF33 antibody we previously prepared. Consistent with our previous observation (Fig. 2), endogenously expressed ORF38 was localized to the cytoplasm (Fig. 4B, column 2). We previously showed that ORF33 was distributed in both the cytoplasm and the nucleus in the absence of other viral proteins (Guo et al., 2009). Here we observed that, in the context of viral infection, endogenously expressed ORF33 accumulated more in the nucleus than in the cytoplasm at 18 h or 24 h postinfection (Fig. 4B, column 3). More interestingly, at these time points, the cytoplasmic portion of ORF33 proteins co-localized with ORF38 (Fig. 4B, column 4).

**Figure 4:**
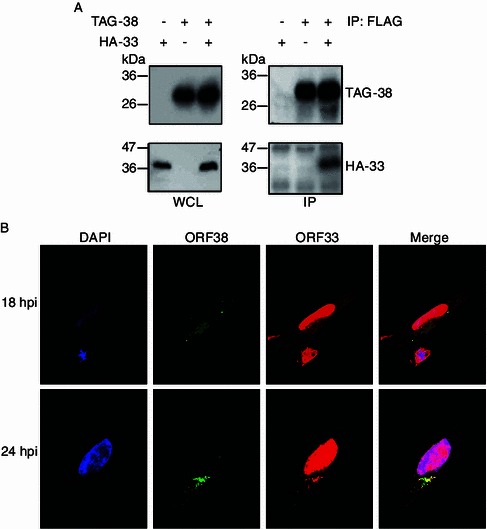
ORF38 interacts with ORF33. (A) ORF38 interacts with ORF33 in transiently transfected cells. 293T cells were transfected with pHA-33 or pTAG-38 or both, and at 48 h posttransfection, whole cell lysates (WCL) were prepared, and the expression of HA-tagged ORF33 and FLAG-tagged ORF38 was examined by Western blotting. The whole cell lysates were further subjected to immunoprecipitation with anti-Flag M2 agarose and analyzed by Western blotting using an anti-HA or an anti-FLAG antibody. (B) Co-localization of ORF38 and ORF33 during virus infection. Vero cells were infected with WT MHV-68 at an MOI of 3. At 24 hpi, cells were fixed for indirect immunofluorescence analysis using an anti-ORF33 antibody and the anti-ORF38 antibody simultaneously

### ORF38 and ORF33 co-localize with TGN

The fact that only the cytoplasmic portion of ORF33 co-localized with ORF38 during viral infection suggested that other viral factors may interact with ORF33 and affect its localization. To focus on the role of ORF38, we further analyzed the interaction of this pair of proteins in the absence of other viral proteins. Cells were transfected with pEGFP-33 or pDsRed-38 or both, stained with DAPI and then observed with a confocal laser microscope. As shown in Fig. 5A, ORF33 by itself localized to both the nucleus and the cytoplasm, whereas ORF38 by itself localized to juxtanuclear space within the cytoplasm. Notably, in the presence of ORF38, almost all ORF33 accumulated in the cytoplasmic areas where ORF38 localized to. Further analysis using TGN38, the protein marker for TGN, demonstrated that the cytoplasmic compartment that ORF33 and ORF38 co-localized with is TGN (Fig. 5B).

**Figure 5:**
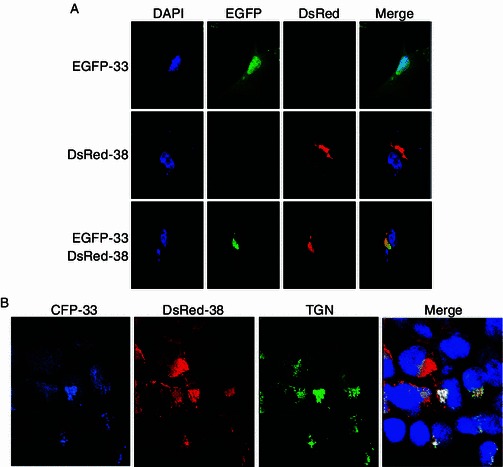
ORF38 and ORF33 co-localize with TGN. (A) Translocation of ORF33 into cytoplasm in transiently transfected cells in the presence of ORF38. HeLa cells were transfected with pEGFP-33 or pDsRed-38 or both. At 24 h posttransfection, cells were stained with DAPI and then observed with a confocal laser microscope. (B) ORF33 and ORF38 co-localize with TGN. 293T cells were co-transfected with pCFP-33, pDsRed-38 and pTGN38-GFP. At 24 h posttransfection, cells were fixed, stained with DAPI, and observed under a confocal laser microscope

## Discussion

Studies from all herpesvirus subfamilies indicated that tegument, composed mostly of tegument proteins, plays a crucial role during virion morphogenesis. Tegumentation is a complicated process that involves multiple protein-protein interactions (Bortz et al., 2007; Guo et al., 2010; Phillips and Bresnahan, 2012; Wang et al., 2012). However, the molecular mechanisms responsible for gammaherpesvirus tegumentation remained unclear. Here we report that MHV-68 ORF38 is a virion protein (Fig. 1B). Furthermore, our trypsin and detergent sensitivity data indicated that ORF38 is a tegument protein but binds to capsid less tightly than ORF33 does, since ORF38 fell off from virions in the presence of detergent only (Fig. 1C). Therefore ORF38 is very likely a component of the outer tegument and associated with glycoprotein(s) in the virion. This idea is consistent with its localization at TGN (Figs. 2B and 5B), where most viral glycoproteins are located.

Tegument formation is generally thought to originate during primary envelopment, with additional tegument proteins being incorporated in the cytoplasm following nuclear egress and finally at the TGN-derived vesicles. Different tegument proteins are added onto virions at different intracellular locations (Kelly et al., 2009). Using the immuno-gold labeling assay, we previously showed that MHV-68 ORF52, a tegument protein essential for virion maturation in the cytoplasm, is present on both immature virions in the cytoplasm and on virions that have entered vesicles (Wang et al., 2012). Unlike ORF52, ORF38 is only present on virions that have entered the vesicles but not present on immature virions outside the vesicles (Fig. 3). Abundant ORF38 is also present on these vesicles where secondary envelopment takes place. Together, these data indicate that ORF38 is packaged into virions during secondary envelopment.

ORF38 is conserved in all three herpesvirus subfamilies. Its homologues in alpha- and beta-subfamily are UL11 and UL99, respectively. UL11 and UL99 are virion associated tegument proteins. Both are myristoylated and accumulate on the cytoplasmic faces of TGN membrane, a site where secondary envelopment occurs to produce infectious virus particles (Sanchez et al., 2000; Loomis et al., 2001). UL11-null mutant of HSV-1 produced less viral particles and non-enveloped nucleocapsids accumulated in the cytoplasm, providing direct evidence that UL11 is involved in the secondary envelopment of virus (Baines and Roizman, 1992). In HCMV, UL99-null mutation also resulted in the accumulation of non-enveloped viral particles in the cytoplasm (Sanchez et al., 2000). More recent data indicated that the interaction between UL99 and UL94 is essential for virion morphogenesis of HCMV in the cytoplasm (Phillips et al., 2012). The ORF38 homologue in EBV, BBLF1, has been identified as a virion associated protein (Johannsen et al., 2004) and proposed to be involved in virion maturation (Chiu et al., 2012). However, the detailed molecular mechanism of ORF38 involved in virion morphogenesis of gammaherpesviruses has remained unclear. Through co-IP and co-localization studies, we showed in this study that ORF38 interacts with ORF33 in MHV-68 not only during transient transfection but also during viral infection. Therefore, the interaction between ORF38 and ORF33 is a conserved event among herpesviruses.

Virion assembly is thought to be driven by multiple protein-protein interactions (Uetz et al., 2006; Rozen et al., 2008; Mettenleiter et al., 2009). After escape from the nucleus, partially tegumented capsids arrive at TGN for secondary envelopment, depending on a number of protein-protein interactions. The interaction between ORF33 and ORF38 is most likely among these interactions for MHV-68. Our previous study using an ORF33-null mutant of MHV-68 showed that ORF33 is not only essential for the cytoplasmic stage of virion maturation but also plays an important role in virion morphogenesis in the nucleus (Guo et al., 2009). Consistently, ORF33 localized to both the nucleus and the cytoplasm during MHV-68 infection (Fig. 4B). Interestingly, in the absence of other viral proteins, ORF33 and ORF38 together mostly localized to TGN (Fig. 5). These observations indicated that interaction of ORF33 with other virion protein(s) may affect its localization during viral infection. Because ORF33 is likely an inner tegument protein (Guo et al., 2009) and ORF38 likely an outer tegument protein (Fig. 1C), we propose that ORF33 may first bind to capsids within the nucleus and through its interaction with ORF38 may assist with the trafficking of nucleocapsids to TGN for final maturation. Further studies are needed to clarify these sequential events.

## Materials and Methods

### Cells and viruses

BHK-21 cells, Vero cells, HeLa cells and 293T cells were cultured in complete Dulbecco’s modified Eagle’s medium (DMEM) supplemented with 10% fetal bovine serum. Wide-type (WT) MHV-68 was propagated by infecting BHK-21 cells at a multiplicity of infection (MOI) of 0.03. To infect BHK-21 cells or Vero cells, the viral inoculum in DMEM was incubated with cells for 1 h with occasional swirling. The inoculum was then removed and replaced with fresh DMEM plus 10% fetal bovine serum. The titer of virus was measured by a plaque assay in BHK-21 cells as previously described (Wu et al., 2000).

### Plasmids construction

The ORF38 expression plasmid with 3xFLAG-CBP epitope, pTAG-38, is a gift from Dr. Ren Sun (University of California, Los Angeles). pTGN38-GFP, pEEA1-GFP, pLAMP1-GFP and pVPS37C-GFP were kindly provided by Dr. Tao Xu (Institute of Biophysics, Chinese Academy of Sciences). Plasmids pHA-33 and pEGFP-33 were constructed in the lab as described previously (Guo et al., 2009). The full-length MHV-68 ORF38 sequence was cloned into pET-30b (Novagen) via the *Eco*RI and *Xho*I sites to construct pET-30b-38 for expression of His-tagged ORF38 in *E. coli* strain Rosetta (DE3). pDsRed-38 was constructed by inserting the full length ORF38 coding sequences into the *Nhe* I and *Xho* I sites of pDsRed2. The sequences of primers used for plasmid construction are available upon request.

### Antibodies, immunoprecipitation and immunoblotting

Purified His-ORF38 of MHV-68 was provided to Experimental Animal Center, Institute of Genetics and Developmental Biology, Chinese Academy of Sciences to generate polyclonal antibodies against ORF38. Polyclonal antibodies for ORF26 and gB were kind gifts from Dr. Ren Sun (University of California, Los Angeles). Polyclonal antibody against ORF33 was described previously (Guo et al., 2009). Mouse monoclonal antibody against the FLAG epitope and the HA epitope were purchased from Sigma. 293T cells seeded onto a 6-cm plate were transfected with 7.5 μg of total DNA by Lipofectamine-2000 (Invitrogen). Forty-eight hours after transfection, cells were washed once with ice-cold phosphate-buffered saline (PBS) and then solubilized in lysis buffer (50 mmol/L Tris-Cl [pH 7.4], 150 mmol/L NaCl, 1% TritonX-100, 1 mmol/L EDTA) with a cocktail of protease inhibitors. Lysates were cleared by centrifugation for 15 min, 13,000 rpm. Ten percent of the supernatant was used as an input control. Soluble proteins were mixed with 20 μL of anti-FLAG M2 agarose (Sigma) and rotated at 4°C for 4 h. Beads were washed three times with lysis buffer prior to use. Immune complexes were washed five times with lysis buffer and supernatant was depleted. Bound proteins were then subjected to immunoblotting analysis. For immunoblotting, samples were heated to 100°C and subjected to SDS-PAGE. Proteins on gels were transferred onto a polyvinylidene difluoride membrane (Millipore) and incubated sequentially with appropriate primary antibody and secondary antibody (anti-rabbit or anti-mouse immunoglobulin G conjugated with horseradish peroxidase), and the proteins were detected by the Enhanced Chemiluminescence system (Millipore).

### Detergent and trypsin treatment assay

MHV-68 virions were purified from the supernatant of MHV-68-infected BHK-21 cells by discontinuous sucrose density gradient ultracentrifugation as described previously (Bortz et al., 2003). Briefly, supernatants were cleared of large debris twice by centrifugation at 4°C and 1000 *g* for 15 min. Extracellular viruses were pelleted by ultracentrifugation through a 7.5% sucrose cushion in a P28S rotor (Hitachi, 4°C, 65,000 *g*, 1 h), resuspended in TN buffer (20 mmol/L Tris [pH 7.8], 50 mmol/L NaCl), and purified by 10% to 45% discontinuous sucrose density gradient ultracentrifugation in a P40T rotor (Hitachi, 4°C, 25,000 *g*, 4.5 h) with slow acceleration and braking. Visible bands between 25%–35% starting sucrose positions was saved. Virions were pelleted by ultracentrifugation in a P40T rotor (Hitachi) at 4°C and 65,000 *g* for 1 h and resuspended in 200 μL of TN buffer. For trypsin and detergent treatment, 20 μL of virion stock was diluted into 200 μL of trypsin buffer (50 mmol/L Tris-HCl [pH 7.5], 150 mmol/L NaCl, 1 mmol/L CaCl_2_) with or without 1% Triton X-100 supplementation, followed by addition of trypsin to a final concentration of 4 μg/mL. After incubation at 37°C for 1 h, the reaction was stopped by adding 0.5 mmol/L phenylmethylsulfonyl fluoride and protease inhibitors, and viral particles were collected by centrifugation at 20,000 *g* for 30 min at 4°C.

### Indirect immunofluorescence assay (IFA)

Vero cells were infected with MHV-68 at an MOI of 3. At 18 h or 24 h postinfection, cells were fixed with 4% paraformaldehyde. The fixed cells were permeabilized with phosphate-buffered saline (PBS) plus 0.2% Triton X-100 and processed for immunofluorescence staining, which was performed using a primary antibody against ORF38 or ORF33. Following incubation with fluorescently labeled secondary antibodies, images were captured with a confocal microscope (Olympus).

### Immunogold TEM

BHK cells were seeded a day before infection with MHV-68 at 50% confluence in 10-cm plates. The cells were infected at an MOI of 5. Sample preparation and immunostaining were performed as described previously, with some modifications (Wang et al., 2012). Briefly, at 11 h after infection, the cells were fixed with 2% paraformaldehyde plus 0.05% glutaraldehyde in 0.1 mol/L phosphate-buffered saline (PBS) (pH 7.2) for 15 min at 4°C on the plate and embedded in 10% gelatin in 0.1 mol/L PBS. Small blocks were infiltrated in 2.3 mol/L sucrose plus 20% polyvinylpolypyrrolidone (PVP) in PBS overnight at 4°C and quickly plunged into liquid nitrogen. Sections approximately 60-nm thick were cut with a Leica UC6/FC6 ultramicrotome and picked up with a wire loop filled with 2.3 mol/L sucrose. Immunostaining was performed as follows: the sections were washed in BSA buffer (1% BSA and 0.15% glycine in PBS) followed by blocking for 30 min using normal goat serum (1:20 dilution in BSA buffer). Subsequently, the sections were incubated for 1 h at room temperature with the anti-ORF38 polyclonal antibody (or rabbit IgG as a negative control) and then for 1 h at room temperature with goat anti-rabbit IgG conjugated with 5-nm colloidal gold particles (Sigma). After a brief wash in BSA buffer and PBS, the sections were treated with 2.5% glutaraldehyde for 5 min. Finally, the sections were stained by 2% neutral uranyl acetate sealed with methyl cellulose and examined with a 120-KV electron microscope (Tecnai; FEI).

## Abbreviation

DMEM: Dulbecco’s modified Eagle’s medium
EBV: Epstein-Barrvirus
HCMV: human cytomegalovirus
HSV-1: herpes simplex virus-1
IFA: indirect immunofluorescence assay
KSHV: Kaposi’s sarcoma-associated herpesvirus
MHV-68: Murine gammaherpesvirus-68
MVB: multivesicular bodies
PBS: phosphate-buffered saline
PVP: polyvinylpolypyrrolidone
TGN: trans-Golgi network
WCL: whole cell lysates
WT: wide-type
